# Membranous location of EGFR immunostaining is associated with good prognosis in renal cell carcinoma

**DOI:** 10.1038/sj.bjc.6601241

**Published:** 2003-09-30

**Authors:** J P Kallio, P Hirvikoski, H Helin, P Kellokumpu-Lehtinen, T Luukkaala, T L J Tammela, P M Martikainen

**Affiliations:** 1 Department of Urology, Tampere University Hospital, PO Box 2000, FIN-33521 Tampere, Finland; 2 Department of Pathology, Oulu University Hospital, PO Box 50, FIN-90029 OYS, Oulu, Finland; 3 Department of Pathology, Tampere University Hospital, PO Box 2000, FIN-33521 Tampere, Finland; 4 Department of Oncology, Tampere University Hospital, PO Box 2000, FIN-33521 Tampere, Finland; 5 Medical School, University of Tampere, FIN-33521 Tampere, Finland; 6 School of Public Health, University of Tampere, FIN-33014 Tampere, Finland

**Keywords:** renal cell carcinoma, epidermal growth factor receptor, prognosis

## Abstract

Epidermal growth factor receptor (EGFR) is a key factor in tumorigenesis. The association between EGFR expression and prognosis in renal cell carcinoma (RCC) is not clear. In our study of 134 RCCs, the cellular location of immunostaining was evaluated and patients with EGFR-positive tumours with prominent membranous staining had a good prognosis. Their overall survival was significantly longer (*P*=0.004) than that of patients with either EGFR-negative tumours or with mainly cytoplasmic staining. However, further studies on the different EGFR expression patterns in RCC are needed to clarify their role in the progression of the disease.

The epidermal growth factor receptor (EGFR) family has been found to play a central role in tumour progression. Ligand binding to the EGFR, receptor dimerisation and the activation of downstream signalling pathways are molecular events involved in tumorigenesis (Kim and Muller, 1999; Carpenter, 2000. High expression of EGFR is considered to be an unfavourable prognostic factor in patients with a variety of tumours (Lieberman *et al*, 1985; Slamon *et al*, 1987; Neal *et al*, 1990), including renal cell carcinoma (RCC) (Uhlman *et al*, 1995; Yoshida *et al*, 1997). However, there are also studies on RCC reporting no association between EGFR and prognosis (Hofmockel *et al*, 1997). In these studies, in addition to ligand binding and immunohistochemical methods (Yoshida *et al*, 1997), also Northern blot (Sargent *et al*, 1989) and Southern blot (Gomella *et al*, 1989) analyses have been used. In the present study, we examined the association between the location of the EGFR immunostaining and prognosis in RCC.

## MATERIALS AND METHODS

### Patients

Our study population consisted of 134 consecutive patients who underwent radical nephrectomy for RCC between 1995 and 1999 at Tampere University Hospital. The median age of the patients (83 men and 51 women) at the time of operation was 64 years (range 35–86 years). Clinical stage was assigned using the TNM Classification of Malignant Tumours (UICC, 1997). The median follow-up time was 40 months, 49 months for survivors and 12 months for nonsurvivors. During the follow-up time, 40 patients died of RCC and 17 of other causes.

### Specimens

Archival formalin-fixed, paraffin-embedded RCC material was used for the study. All tissue blocks were re-evaluated (PH), and from a representative area for each tumour a 3-mm core was transferred to a multi-tissue block, which was then used for further analysis. All tumours were classified according to Heidelberg classification (Kovacs *et al*, 1997) and graded according to the Fuhrman system (Fuhrman *et al*, 1982) by two pathologists (HH, PM).

### Immunohistochemical staining

Paraffin-embedded multi-tissue blocks were cut 4–5 *μ*m in thickness and mounted on precoated slides. After deparaffinisation, antigen retrieval was performed by heating the sections in a microwave oven for 2 × 7 min in 10 mM Tris/1 mM EDTA (pH 9.0) buffer, followed by washes with water. A polyclonal rabbit anti-EGFr variant III antibody (Zymed Laboratories, Inc., San Francisco, CA, USA) was used for EGFR immunostaining at a concentration of 5 *μ*g ml^−1^. A TechMate™ 500 Plus Immunostainer (DAKO a/s, Glostrup, Denmark) was used for the staining procedure and a ChemMate™ peroxidase/DAB detection kit (DAKO a/s, Glostrup, Denmark) for visualisation of the antigen–antibody complex. Sections were slightly counterstained with haematoxylin.

### Evaluation of immunohistochemical staining

**Figure 1:**
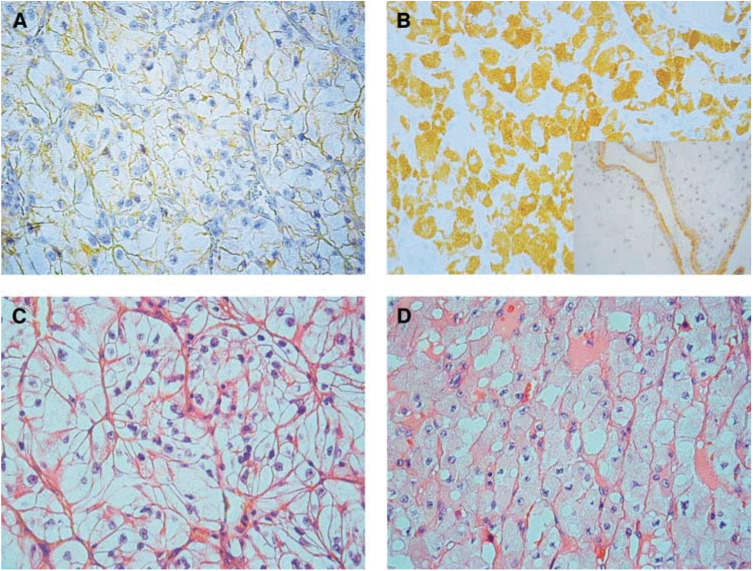
Membranous (**A**) and cytoplasmic (**B**) EGFR staining of two cases of RCC and the corresponding morphology (**C**, **D**) in haematoxylin and eosin staining. Placental control of EGFR staining is shown in the inset in **B**.

The optimal titre for EGFR staining was defined as the dilution giving clearly identifiable membrane staining and negligible background on human placental samples. The intensity of the immunostaining in RCC (scale 0–3) was multiplied by the percentage of cells with positive staining to give a score of 0–300. Thus scored, the positive placental control (Neal *et al*, 1990) in our system gave a score of 100 (Figure 1

, inset). Five staining patterns were scored: solely (m) or predominantly (m>c) membranous staining, solely (c) or predominantly (c>m) cytoplasmic staining or equal (c=m).

### Statistical analysis

Associations of different staining patterns with other main prognostic parameters were tested using Fisher's exact test. Cox regression analysis was used to test for differences in RCC-specific survival. Both univariate and multivariate analyses were performed. In multivariate analysis, prognostic significance was tested for age at operation time, TNM stage, Fuhrman grade, gender and location of EGFR staining. Deaths from causes other than RCC were considered as censored events. An SPSS software version 11.0 was used for statistical analyses.

### Ethics

The research plan was approved by the Ethical Committee of Tampere University Hospital, and oral informed consent was obtained from every patient.

## RESULTS

Altogether, 69 of the tumours (52%) were local (T1–2, N0, M0), 27 (20%) locally advanced (T3–4, N0, M0) and 38 (28%) disseminated (T1–4, N+/M+); 122 (89%) of the tumours had conventional type (clear cell), six (5%) papillary, two (2%) chromophobe, two (2%) collecting duct histology. Two of the tumours (2%) remained unclassified. By nuclear grading, four (3%) of the tumours were classified as grade 1, 59 (44%) as grade 2, 57 (43%) as grade 3 and 14 (10%) as grade 4.

Epidermal growth factor receptor scores varied between 0 and 300, mean score was 83. In all, 73% of tumours were positive in EGFR immunostaining with cutoff score 20. When placental control (score 100) was used as a cutoff score, the percentage of positive tumours was 49%. Papillary tumours had markedly elevated scores (mean 140±63) when compared to those with clear cells (mean 79±69). The distribution of EGFR immunostaining was as follows: no staining 23%, m 3% (Figure 1A), m>c 11%, m=c 13%, c>m 25% and c 25% (Figure 1B). Stratification into three groups was carried out: 1: no staining (23%), 2: prominent membranous staining (27%, including cases m, m>c and m=c) and 3: predominantly cytoplasmic staining (50%, including cases c>m and c). Papillary tumours all had cytoplasmic staining.

**Figure 2:**
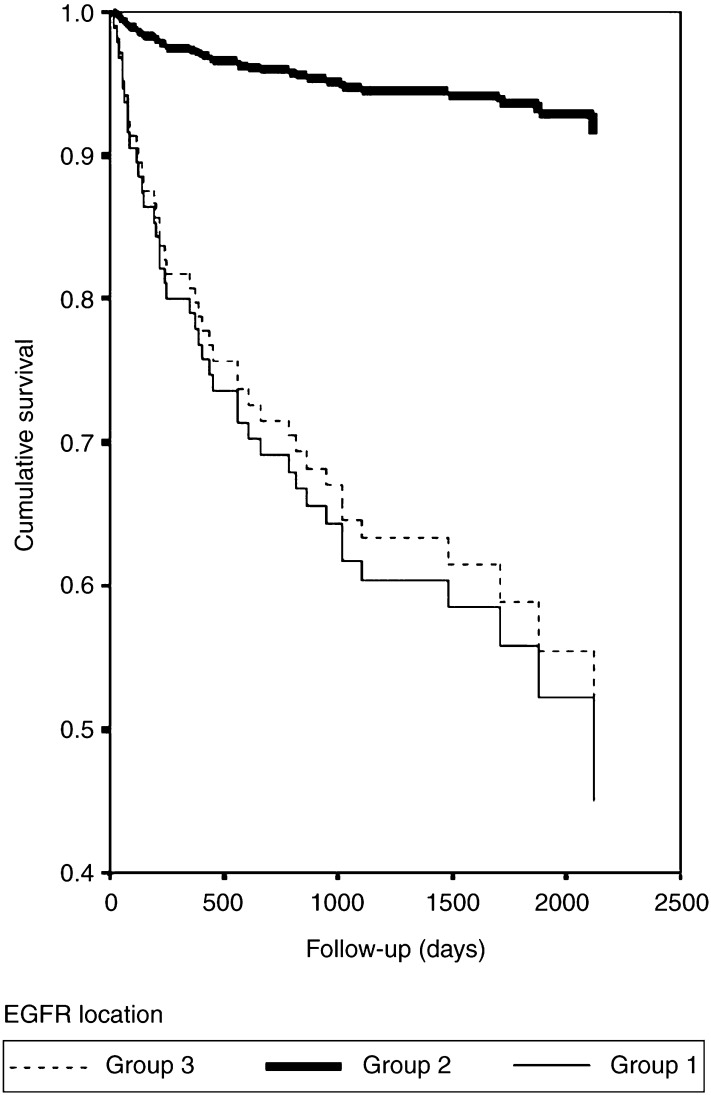
Survival of RCC patients and its correlation with EGFR localisation. Group 1: no staining, group 2: prominent membranous staining and group 3: predominantly cytoplasmic staining.

In univariate analysis for survival, predominantly membranous staining (Group 2 in Figure 2

) associates with good prognosis, (hazard ratio (HR) 8.0; 95% CI 2.0–33.2; *P*=0.004). When EGFR expression was handled as a continuous numeric factor (score) or divided into two classes (cutoff score 20 or 100), there was no statistically significant association with RCC survival. In the multivariate analysis, TNM stage was a very strong and single significant prognostic indicator (HR 37.0; 95% CI 8.2–167.5; *P*<0.001). The membranous immunostaining was not associated with low stages 1–2 (*P*=0.091, Table 1

**Table 1:** Crosstabulated EGFR immunostaining location classes *vs* stages

	**Stage**			
**EGFR staining location**	**1**	**2**	**3**	**4**
No staining	11	4	5	11
	20.4%	26.7%	17.9%	29.7%
Membranous staining	19	12	11	4
	35.2%	13.3%	39.3%	10.8%
Cytoplasmic staining	24	9	12	22
	44.4%	60.0%	42.9%	59.5%
	100%	100%	100%	100%

No statistical associations, *P*=0.091, Fisher's exact test.

), but there was a statistically significant association with low Fuhrman grades (*P*=0.001, Fisher's exact test, Table 2

**Table 2:** Crosstabulated EGFR immunostaining location classes *vs* Fuhrman nuclear grades

	**Fuhrman nuclear grade**			
**EGFR staining location**	**1**	**2**	**3**	**4**
No staining	0	11	15	5
	0.0%	18.6%	26.3%	35.7%
Membranous staining	3	25	6	2
	75.0%	42.4%	10.5%	14.3%
Cytoplasmic staining	1	23	36	7
	25.0%	39.0%	63.2%	50.0%
	100%	100%	100%	100%

The membranous immunostaining has a statistically significant association with low Fuhrman grades, *P*=0.001, Fisher's exact test.

).

## DISCUSSION

In the present study, we showed the importance of the location of EGFR immunostaining in evaluating the prognosis in an unselected group of patients treated for RCC. The study was carried out on unselected consecutive patients operated for RCC in a prospective manner having a well-defined follow-up schedule.

High expression of EGFR has been associated with advanced stage, poor prognosis and high metastatic potential in many human tumours (Lieberman *et al*, 1985; Slamon *et al*, 1987; Neal *et al*, 1990). The association between EGFR expression and prognosis in RCC has not been established. There are several complicating factors: the histological heterogeneity of RCC and the individual properties of different assessment methods. For example, the ligand binding method, which has been held to be a sensitive method for measurement of EGFR, measures functional properties and the technique concentrates on membranous proteins only (Yoshida *et al*, 1997). Immunohistochemistry, on the other hand, offers a simple and economical means for cellular detection of EGFR and analysing its location in tumour cells. So far, several immunohistochemical studies have shown that positive EGFR staining in RCC is common and is associated with cell proliferation (Moch *et al*, 1997), but its role as a prognostic factor remains uncertain (Hofmockel *et al*, 1997). In the immunohistochemical study by Uhlman *et al* (1995), membranous EGFR expression was associated with high tumour grade, metastatic disease and poor disease-specific survival. In contrast to that, in our study membranotic positivity was associated with good prognosis, while cytoplasmic or negative EGFR staining was not. The explanation for this discrepancy is unclear. However, in the present study, both the intensity and location of EGFR staining in tumour cells were taken into account when evaluating its associations with prognosis, while in the study by Uhlman *et al*, there is no description of staining pattern and differences in antibody and staining process may partly explain different results. A similar adverse prognostic role of cytoplasmic EGFR staining demonstrated in our study has also been shown in squamous cell carcinoma of the lung (Piyathilake *et al*, 2002). The association between poor prognosis and cytoplasmic EGFR staining may be due to changes in ligand–EGFR complex internalisation and activation of associated signalling pathways in the progression of RCC, the theory that Piyathilake *et al* have further strengthened by cell culture experiments. The localisation pattern of immunostaining has also been shown to have prognostic value with other markers in cancer like bcl-2 expression in malignant melanoma. In studies by Vlaykova *et al* (2002), a diffuse localisation of bcl-2 expression by immunostaining was associated with better survival than negative or focal expression in malignant melanoma. Similarly, the aberrant cellular location of some adhesion molecules such as alpha-catenin may result in tumour dedifferentiation and aggressive, metastatic phenotype (Hirvikoski *et al* (1998) in laryngeal carcinoma). In the present study, the presence of immunostaining in the membranes was associated with exceptionally good prognosis. It can be speculated that in RCCs in which EGFR distribution in cell membranes is maintained, the growth is probably still controlled by EGF rather than by activation of new signalling pathways. Thus, the distinction between two different survival groups within the EGFR-positive RCCs, in addition to being a candidate for a simple prognostic marker, opens up the challenging possibility of different molecular targets for drug development for these patient groups. The third group in our study, RCCs with totally negative EGFR staining, may include a group of cancers in which mechanisms other than EGF are responsible for cancer growth and progression.

In our study, prominent membranous EGFR immunostaining associates with good prognosis in RCC. However, further studies will be needed to clarify the role of the different EGFR patterns in development and progression of RCC.
